# Study of Applying Naturally Occurring Mineral Materials for Silicone Pressure-Sensitive Adhesives

**DOI:** 10.3390/ma16052092

**Published:** 2023-03-03

**Authors:** Adrian Krzysztof Antosik, Edyta Kucharska, Karolina Mozelewska

**Affiliations:** Department of Chemical Organic Technology and Polymeric Materials, Faculty of Chemical Technology and Engineering, West Pomeranian University of Technology, 70-322 Szczecin, Poland

**Keywords:** functionalized palygorskite, grafting of 3-mercaptopropyltrimethoxysilane, filler modification, new self-adhesive tapes

## Abstract

Silicones are commonly used as adhesives when high-quality materials are required due to harsh environmental conditions such as high temperature, humidity, etc. To ensure high resistance to environmental conditions, including high temperatures, modifications of silicone adhesives are made using fillers. The characteristics of a modified silicone-based pressure-sensitive adhesive with filler are the focus of this work. Functionalized palygorskite was prepared in this investigation by grafting 3-mercaptopropyltrimethoxysilane (MPTMS) onto palygorskite (palygorskite-MPTMS). The palygorskite was functionalized using MPTMS under dried conditions. FTIR/ATR spectroscopy, thermogravimetric analysis, and elemental analysis were all used to characterize the obtained palygorskite-MPTMS. MPTMS loading onto palygorskite was also proposed. The results demonstrated that palygorskite’s initial calcination favors the grafting of functional groups on its surface. New self-adhesive tapes based on palygorskite-modified silicone resins have been obtained. This functionalized filler allows for the improvement of the compatibility of palygorskite with specific resins for application in heat-resistant silicone pressure-sensitive adhesives. The new self-adhesive materials showed increased thermal resistance while maintaining good self-adhesive properties.

## 1. Introduction

Pressure-sensitive adhesives constitute a wide group of industry materials used in industry and households. Their application is very wide: from applications in medicine for the treatment of wounds, as soft and viscoelastic, and at the same time solid (to adapt to human skin), to applications in heavy industry, where high adhesion, cohesion, and resistance to weather conditions are essential [1].

Pressure-sensitive adhesives (PSAs) are polymeric materials that are permanently sticky at room temperature and adhere to surfaces under low pressure using noncovalent forces [2]. PSA differ from other types of adhesives in that they stick when solid (they are not liquid when applied, do not solidify when chemically reacted, and are not hot melt adhesives that are sticky when melted and harden when cooled to room temperature) [2,3]. Pressure-sensitive adhesives are special materials, which should exhibit various properties: high intermolecular mobility, cohesive strength, long relaxation time, and resistance to aging [4]. Currently, the most commonly used monomers are styrene-isoprene-coplymersstyrene–isoprene–styreneylic copolymers; styrene–isoprene–styrene block copolymers (SBCs); styrene-butadiene—SBCs, styrene-butadiene rubbers, polyesters, natural rubbers, and polysiloxanes [3,5]. PSA made from these chemical families already have an established position in the self-adhesive materials market. Recently, there have also been reports of adhesives made from raw materials derived from biological sources (renewable), as an alternative to adhesives made from petrochemical raw materials [3,6].

Silicones are one of the commonly used groups of pressure-sensitive adhesives. Silicone PSAs are nonstoichiometric silicone elastomers with a low degree of cross-linking (they remain close to their gelling threshold). Currently, they are the preferred adhesive system for special applications due to their adhesive properties (e.g., to the skin) and high cohesion. They are also characterized by a low surface tension, which facilitates the mobility of the polymer chains on the surface, thus creating a large contact area on an uneven surface. In addition, silicone adhesives, when removed, do not leave any traces on the surface, they can be easily removed [5,7].

Palygorskite is a nonmetallic mineral composed of hydrated magnesium and aluminum. Its crystalline structure is porous and consists of tetrahedral layers alloyed together along longitudinal sideline chains [8,9,10]. Owing to its large specific surface area and high thermal stability, this inexpensive and readily available mineral finds many applications [9,10]. In mineralogy, palygorskite is classified as a sepiolite due to its similar microscopic structure and morphology to that of sepiolite. The ideal palygorskite crystal is the trioctahedral counterpart to the magnetic and dioctahedral sepiolite [11,12,13,14]. Owing to its unique structure and considerable textual properties, natural palygorskite is a very promising filler [15,16,17]. In addition, the functionalization creates surface functional groups on this natural silicate that readily react with resin, strengthening the interfacial bonds between the resin and palygorskite, and thereby increasing the thermal resistance of the silicone adhesives obtained [18,19,20,21,22]. Studies by other authors have shown that functionalization of the surface of materials with a thiol silane coupling agent (3-mercaptopropyltrimethoxysilane) makes it possible to obtain modified materials containing active thiol groups (–SH), increasing their adhesion. In addition, the availability of active centers (–SH) of MPTMS functionalized materials is one of the parameters enabling their use as fillers for silicone adhesives [23,24,25,26,27,28].

One strategy for improving the interfacial adhesion between the filler and the polymer matrix is to chemically functionalize natural materials (for example, sepiolite, montmorillonite, attapulgite, or bentonite) with coupling agents (organosilanes). The most commonly used coupling agent is 3-aminopropyltriethoxysilane (APTES) [23,24,25,26,27,28]. Our previous studies on the functionalization of porous materials have shown that excessive humidity limits the coupling agents (mainly MPTMS) for these materials. In the presence of moisture, siloxanes condense to polysiloxanes, which limits the action of coupling agents [29]. This has given rise to the development of an effective method for the functionalization of natural porous materials using MPTMS.

The paper shows that the surface modification of calcined palygorskite with MPTMS leads to obtaining a chemically active material [23]. Hydroxyl groups present on the surface of the functionalized material react with the methoxy groups of 3-mercaptopropyltrimethoxysilane, allowing MPTMS to anchor on the palygorskite surface [30,31,32,33]. Recently, silica materials have been increasingly used as fillers for silicone pressure-sensitive adhesives [34,35,36,37,38,39]. To the best of our knowledge, the use of a palygorskite AGLEV^®^SI 200 functionalized 3-mercaptopropyltrimethoxysilane (MPTMS) for modifying silicone pressure-sensitive adhesive has not yet been reported.

This paper aims to develop an effective method of MPTMS linking on the surface of the palygorskite, and to present the study of applying modified materials for silicone pressure-sensitive adhesives to obtain new self-adhesive materials with increased thermal resistance. In this paper, the preparation of modified palygorskite-MPTMS is demonstrated. The possible proposed mechanism of MPTMS grafting onto the surface of palygorskite is discussed. The use of modified palygorskite fillers as additives for silicone pressure-sensitive adhesives has not been previously described. We found that the introduction of modified palygorskite fillers increases the maximum operating temperature of the adhesive, making them a potential candidate for use as adhesives for specialized applications when high temperatures are present. Palygorskite-MPTMS have not been studied as materials for new self-adhesive tapes based on palygorskite-modified silicone resins [40,41,42,43,44,45,46,47]. For this reason, we investigated their potential application in silicone pressure-sensitive adhesives. Natural porous materials functionalized with coupling agents have found wide application in many branches of industry and environmental engineering.

## 2. Materials, Chemicals, and Methods

### 2.1. Materials

Dow Corning (Midland, MI, USA) provided the silicone resin (Dowsil 2013 adhesives) and TERRA ENEO provided the palygorskite AGLEV^®^SI 200 (Luszowice, Poland).

### 2.2. Chemicals

3-Mercaptopropyl-trimethoxysilane (MPTMS, 95%) was purchased from Chemat (Konin, Poland). Chloroform was supplied by Chempur (Poland), and toluene was supplied by Carl Roth (Karlsruhe, Germany). SYL-OFF 4000 was supplied by Dow Corning (USA). All reagents were of analytical grade.

### 2.3. Functionalization of Palygorskite with MPTMS

Two hours were spent calcining the palygorskite AGLEV^®^SI 200 at 500 °C. Dry conditions were used for the incorporation of 3-mercaptopropyl-trimethoxysilane (MPTMS) into calcined palygorskite. In a 250 mL glass reactor, 10 g of calcinated palygorskite was combined with 0.5, 1.0, and 1.5 g of MPTMS and 150 mL of chloroform. The reaction mixture was stirred (with a magnetic stirrer) for 24 h at room temperature, then washed with 100 mL of chloroform and filtered on a pressure funnel using a Whatman paper filter. It was dried in an oven for the next 24 h at 80 °C.

The dried functionalized palygorskite was ground and then sieved through a mesh. The samples are denoted as follows: palygorskite-0.5MPTMS, palygorskite-1.0MPTMS, and palygorskite-1.5MPTMS. After modification of palygorskite, the samples were characterized by FTIR/ATR, TGA (for thermal stability), and elemental analysis. Moreover, to study the applicability of the functionalized material, the use of the silicone pressure-sensitive adhesive as a filler was performed.

### 2.4. Preparation of Si-PSA Modification Tape

To obtain the composition of the modified adhesives, the platinum catalyst Syl-OFF 4000 in the amount of 1.5 parts was added to the commercial resin (DOWSIL 2013) and mixed until homogeneous. Then, 0.1, 0.5, 1, or 3 wt.% based on the weight of the resin filler was added and mixed until a homogeneous composition was obtained. These compositions were coated onto a 50 μm thick polyester film using a Byk slit applicator (ALTANA AG, Wesel, Germany) to obtain an XY thick adhesive film, which was introduced into a Binder dryer (Binder GmbH, Tuttlingen, Germany) to cross-link according to the catalyst safety data sheet (temperature of 150 °C; time 15 min). This adhesive film was secured with another layer of polyester film.

### 2.5. Fourier Transform Infrared

FTIR spectra were recorded using a THERMO NICOLED 380 apparatus (Waltham, MA, USA) equipped with an ATR diamond plate. Thirty-two scans were acquired in the 4000–400 cm^−1^ range with a resolution of 4 cm^−1^.

### 2.6. Thermogravimetric Analysis

TGA was carried out using a thermomicrobalance from NETZSCH (Selb, Germany) with a scan ranging from 25 to 800 °C at a constant heating rate of 10 °C/min, in an air atmosphere with nitrogen flow as the purge gas. Samples with mass 9–10 mg were loaded into an Al_2_O_3_ crucible.

### 2.7. Elemental Analysis

The elemental analysis CHNS of calcined palygorskite, palygorskite-0.5MPTMS, palygorskite-1.0MPTMS, and palygorskite-1.5MPTMS was performed by using a Thermo Scientific™ FLASH 2000 CHNS/O Analyzer (Waltham, MA, USA). Samples about 2.8 mg were weighed to an accuracy of tin crucibles.

### 2.8. Pot-Life

Pot-life is a value that indicates the pot-life of the adhesive composition after modification for use (for coating). It is described as the time needed to double (for higher viscosity) or quadruple (for low viscosity) the viscosity of the original mixture. The test is carried out at room temperature (23 °C) and the measurement is started immediately after mixing the composition [48].

### 2.9. Adhesion

The peel adhesion of pressure-sensitive silicone adhesives was tested using a Zwick-Roell Z1 machine by applying the procedures described by the Association des Fabricants Europeens de Rubans Auto-Adhesifs (AFERA) 4001 international standard. A 2.5 cm wide adhesive tape was stuck to a steel plate under pressure from 2 kg of a hard rubber roller. The plate was placed in the jaws of a testing machine and the free end was placed into the other jaw. The test measures the force with which the tape peels off the plate at a constant speed of 300 mm/min. The result is the average of three measurements [48,49].

### 2.10. Cohesion

The study of the properties of cohesion and the adhesive–cohesive balance was conducted in three ways. In the first and second tests, the tests were carried out at room temperature and elevated temperature (70 °C), respectively, according to the AFERA 4012 standard. In this method, a 2.5 cm × 2.5 cm sample of the tape is glued to a metal plate and then loaded with a 1 kg weight. During the test, the time taken for the tape sample to detach from the metal plate is measured [50,51].

Lastly, the shear adhesion failure test (SAFT) was carried out to determine the Si-PSA resistance to elevated temperature. For this purpose, the samples prepared for the cohesion test were placed in the shear tester designed at ILSAM (West Pomeranian University of Technology in Szczecin). This apparatus was developed to determine the temperature and time in which the weld will break. The test was carried out in the range 20–250 °C with a temperature increase of 0.5 °C min^−1^ [52].

### 2.11. Tack

The tack was also measured using a Zwik-Roell Z1 testing machine according to the procedures described by AFERA 4015. This method measures the force to detach the tape from a metal plate without pressure. The contact area of the adhesive layer with the substrate during the test was 5 cm^2^ (2.5 cm × 2 cm) [36,53].

### 2.12. Shrinkage

The shrinkage of the cross-linked pressure-sensitive adhesives was investigated using the cross-cut method. The test is carried out with the use of PVC or PET foil, covered with an adhesive, which is stuck to metal. Then, the prepared plate is placed at a temperature of 70 °C and after a specified time (10 and 30 min; 1, 3, 8, and 24 h; 2, 3, 4, 5, 6, and 7 days), the change in film size (width of the slits formed by the cuts) is examined. The test result is the arithmetic mean of eight points [36,52].

## 3. Results and Discussion

### 3.1. Fourier Transform Infrared (FTIR/ATR)

**Figure 1:**
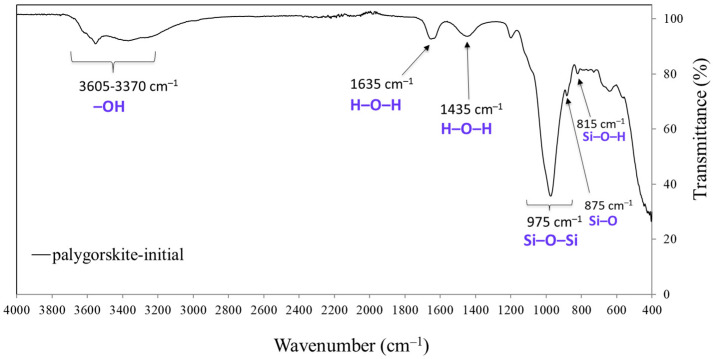
Fourier transform infrared (FTIR) spectra of initial palygorskite—black line.

Figure 1 shows the FTIR spectra of the initial palygorskite.

To learn more about the surface properties of raw palygorskite, FTIR analyses were carried out. Main absorption bands for palygorskite before calcination (initial palygorskite, Figure 1) were found to be at 815, 875, 975, 1435, 1635, 3605–3370 cm^−1^. Both the Si–O–H vibration at 815 cm^−1^ and the Si–O stretching of the silanol group are reflected in the 875 cm^−1^ band. Owing to the stretching vibrations of the Si–O–Si siloxane groups, the 975 cm^−1^ band is very prominent. Water’s H–O–H bending vibration is reflected in the bands at 1435 and 1635 cm^−1^. Internal silanol causes bands at 3605–3370 cm^−1^ due to strongly interacting vicinal –OH [29,54,55,56].

**Figure 2:**
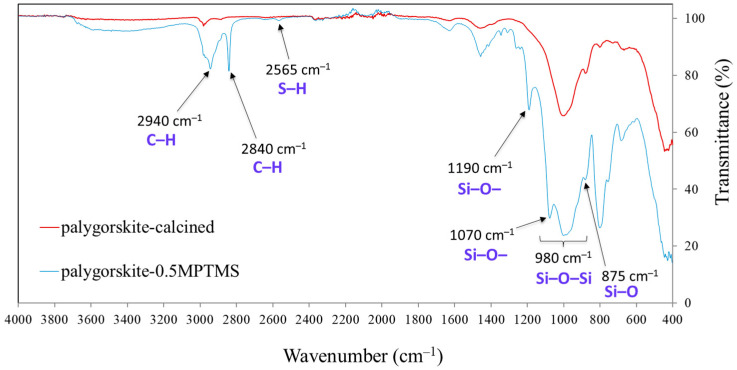
Fourier transform infrared (FTIR) spectra of palygorskite after calcination (red line) and palygorskite after functionalization (palygorskite-0.5MPTMS) (blue line).

Figure 2 shows the FTIR spectra of the example palygorskite functionalized with 3-mercaptopropyltrimethoxysilane and palygorskite after calcination.

As a result of the adsorptive evaporation of moisture occurring during the calcination process, the amount of hydroxyl groups –OH (at 1435, 1635, 3605–3370 cm^−1^) on the surface of porous materials can be reduced (palygorskite-calcined—red line, Figure 2). MPTMS grafting to palygorskite is dependent not only on the pore structure of palygorskite but also on the amount of surface functional groups [29,54,55,56].

In the palygorskite after functionalization of 3-mercaptopropyl-trimethoxysilane (palygorskite-0.5MPTMS, blue line—Figure 2), a band at 2565 cm^−1^ indicative of the presence of a thiol group (–SH) were observed. The two sharp peaks at 2940 and 2840 cm^−1^ come from the asymmetric –CH stretching vibration. There is also observed the presence of a band at 980 cm^−1^, which is associated with Si–O–Si bonds. In the palygorskite after functionalization, the peaks at 1190 and 1070 cm^−1^ come from the Si–O– linkages, which further support the successful modification of the AGLEV^®^SI 200. In the case of palygorskite-1.0MPTMS and palygorskite-1.5MPTMS, FTIR spectra of these samples also revealed the presence of the mentioned bands [29,54,55,56].

### 3.2. Thermogravimetric Analysis

**Figure 3:**
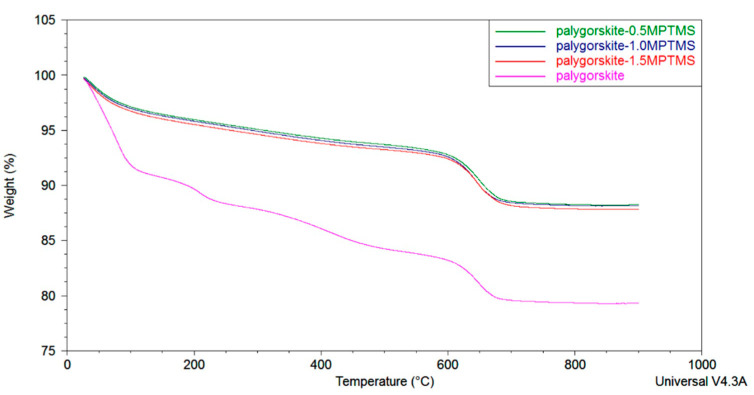
TGA diagrams of pure and functionalized by MPTMS palygorskite.

The thermograms of pure palygorskite and palygorskite modified with 3-mercaptopropyl-trimethoxysilane (MPTMS) are shown in Figure 3.

Figure 3 shows TGA diagrams of pure and functionalized using MPTMS palygorskite. The TGA curves were similar for all modified samples. Weight loss was greater for the unmodified palygorskite, and four distinct stages of degradation were observed. The thermal decomposition occurs in the intervals 75–125 °C (I), 200–250 °C (II), 300 and 400 °C (III), and 550 and 700 °C (IV). The initial mass loss is caused by the release of bound water and occurs quickly. Owing to the reversible nature of the phenomenon and the clay’s excellent absorption properties, the second water loss is equivalent to the complete disappearance of the water. As the structure changes towards a rafter structure, water (coordinated to cations located along octahedral sheets) is lost, accounting for the third weight loss. Note that even in this condition, palygorskite can still absorb water via capillarity. The dihydroxylation of Mg-OH groups in palygorskite results in the loss of a fourth water molecule from the mineral’s overall water mass. The only exothermic peak at around 550–700 °C in the case of the functionalized palygorskite, could be assigned to the decomposition of silane grafting [29,56]. In temperatures above 500 °C, the carbonates in palygorskite begin to decompose, releasing CO_2_ [57,58,59,60,61]. TGA analysis revealed an increase in thermal stability between the pure and modified palygorskite. It has been suggested that the decomposition and oxidation of MPTMS incorporated into palygorskite accounts for the exothermic peaks and ignition losses around 550–700 °C. The silane is strongly bonded onto the palygorskite surface; silane is not removed by washing or heating (Figure 3) [61,62,63].

### 3.3. Elemental Analysis

The presence of thiol groups in modified palygorskite was confirmed by elemental analysis (Table 1).

The elemental analyst data provide evidence that pure palygorskite does not contain sulfur (S = 0.00 ± 0.00). Thiol groups (–SH) have been observed in the palygorskite functionalized with 3-mercaptopropyltrimethoxysilane (palygorskite-0.5MPTMS, palygorskite-1.0MPTMS, and palygorskite-1.5MPTMS). The elemental analysis showed that the sulfur content of the samples was, respectively, 0.44, 0.42, and 0.42% (Table 1).

The reaction efficiency was also confirmed by elemental analysis (Table 1). As can be seen, the highest efficiency of the reaction (66.00% ± 0.00) when carrying out the modification was obtained using MPTMS at 0.5 wt.% in relation to the amount of palygorskite (paligorskite-0.5MPTMS). A two-fold and three-fold increase in the amount of MPTMS did not increase the sulfur content of the modified material (1.0 wt.% MPTMS in the case of palygorskite-1.0MPTMS and 1.5 wt.% MPTMS in the case of palygorskite-1.5MPTMS were used) (Table 1).

**Scheme 1:**
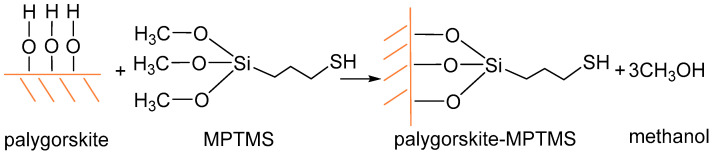
A chemism for a 3-mercaptopropyl-trimethoxysilane reaction with a palygorskite surface [23,38].

We propose a chemism for a 3-mercaptopropyl-trimethoxysilane reaction with a palygorskite surface (Scheme 1). To prevent condensation of MPTMS to reactive silanols, the functionalization of calcined palygorskite is carried out under anhydrous conditions (in a chloroform environment). Methoxy groups (–OCH_3_) derived from 3-mercaptopropyl-trimethoxysilane (MPTMS) react with hydroxyl groups (–OH) found on the surface of palygorskite, thus a functionalized material palygorskite-MPTMS (according to the scheme shown below) is obtained. The result of successful functionalization is the methanol formed in the process. The presence of the formed methanol was confirmed by GC-MS analysis (Figures S1 and S2) [23,38].

The findings of Khieu et al. [23] show that when exposed to water, silane molecules become highly hydrated and condense into each other rather than remaining attached to the material wall. Condensation of silanols during functionalization leads to the formation of siloxane bridges, which are unfavorable for coupling reactions [23,42].

The findings of Khieu et al. [29] show that when exposed to water, silane molecules become highly hydrated and condense into each other rather than remaining attached to the material wall. Researchers have found that MPTMS incorporation is less likely to occur when materials are dehydrated prior to silane treatment [64]. Assigning the exothermic peak at that time, roughly 300 °C, to the breakdown of silanes [64], is a reasonable hypothesis. Condensation of silanols during functionalization leads to the formation of siloxane bridges, which are unfavorable for coupling reactions.

**Figure 4:**
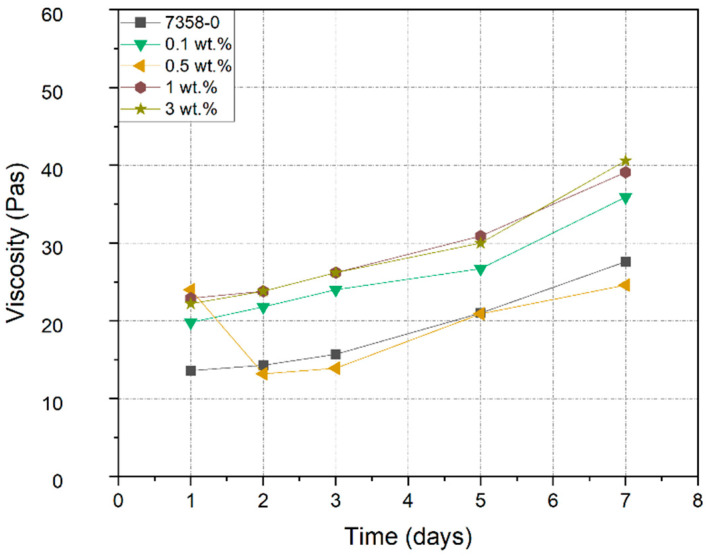
Influence of palygorskite addition on Si-PSA composition viscosity.

**Figure 5:**
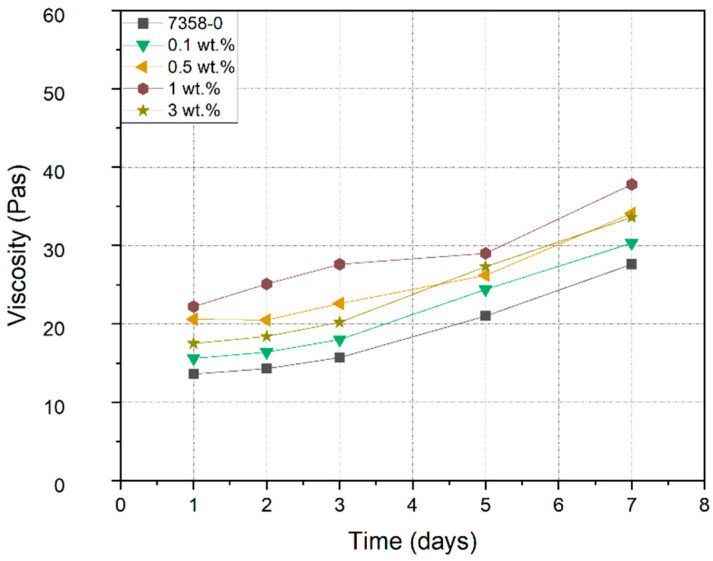
Influence of palygorskite-0.5MPTMS addition on Si-PSA composition viscosity.

**Figure 6:**
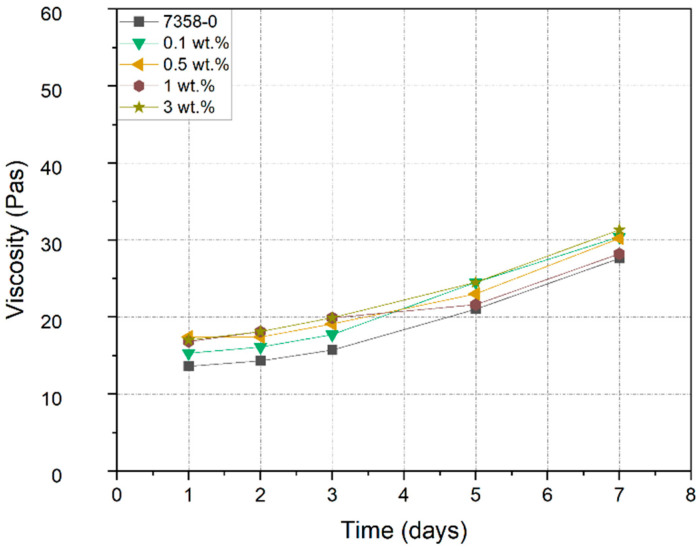
Influence of palygorskite-1.0MPTMS addition on Si-PSA composition viscosity.

**Figure 7:**
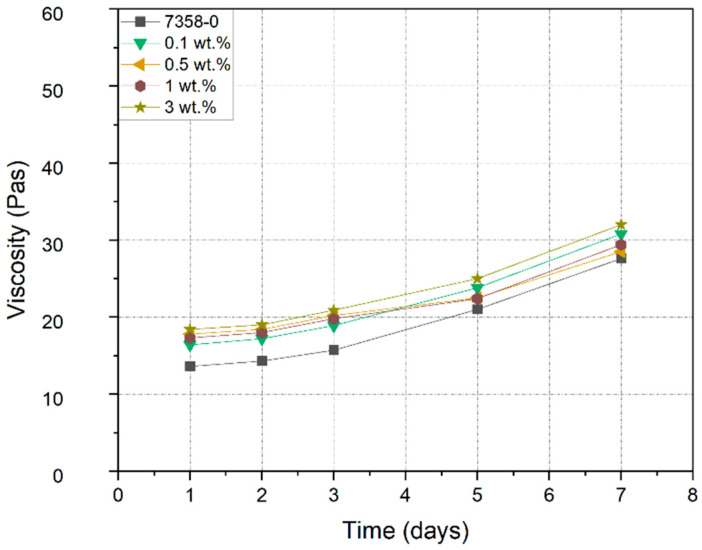
Influence of palygorskite-1.5MPTMS addition on Si-PSA composition viscosity.

Figure 4, Figure 5, Figure 6 and Figure 7 showed the influence of filler and modified filler on viscosity in time-prepared Si-PSA composition. Nonmodified palygorskite exhibits increased viscosity for a small amount (0.1 wt.%), then a decrease for 0.5 wt.%, below the nonfiller formulation value, followed by a further increase. This may be due to the effect of the better ordering of polymer chains by the addition of small amounts of filler (0.1 wt.%), and its disappearance by good dispersion of the filler in the resin for fillings up to 0.5. wt.%. Despite this, this amount of filler behaves as spatial impediments (despite good dispersion and lack of agglomeration), interfering with the orientation of the polymer, which results in a decrease in viscosity. In the case of higher fillings, the amount of filler addition is associated with its agglomeration. (For both 1 and 3 wt.%, the viscosity values were very similar). Viscosity changes were noted for both pure palygorskite and its modifications. Each modification resulted in obtaining higher viscosity values with increasing filler content in the adhesive composition. On the other hand, the greater the amount of the filler-modifying compound, the closer to each other the curves of successive filling of the composition are, and their values do not differ so much from each other. An increase in the viscosity of the composition through time was noted in all of the tested combinations (adhesive compositions behave as thixotropic liquids); in most cases, it was a two-fold increase in the initial value, which in practice makes it impossible to use such a composition for coating. The increase in adhesive viscosity through time can be explained by the significant copolymer–filler interaction resulting from the high surface energy of pure filler together with interactions between silicone adhesive and filler.

**Figure 8:**
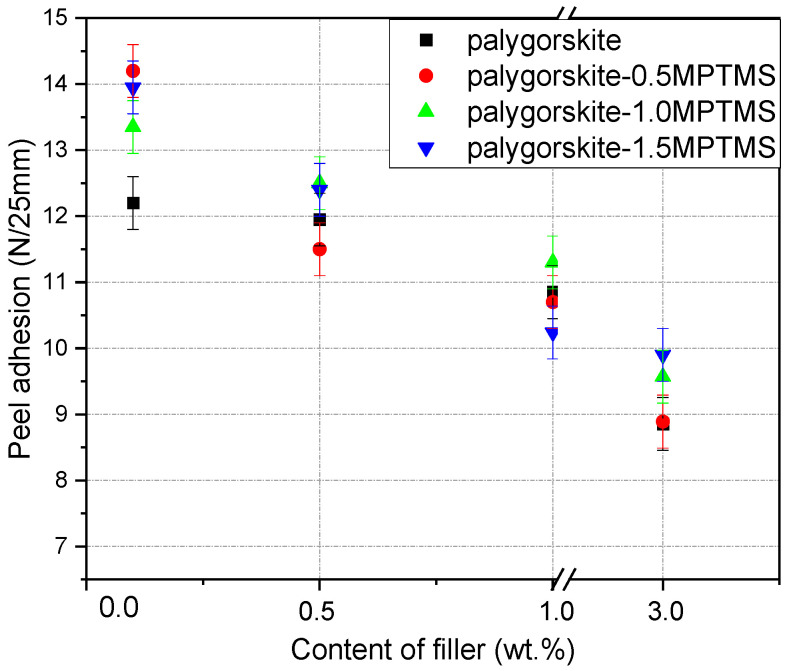
Influence of palygorskite modified by MPTMS addition on Si-PSA tape adhesion.

The influence of the addition-modified palygorskite by MPTMS on adhesion silicone pressure-sensitive adhesives is shown in Figure 8. In all tested cases, increasing the filler addition resulted in a decrease in the adhesion value, which is consistent with other examples of modification with fillers, e.g., montmorillonite, described in the literature [36,52,59]. An increase in the amount of filler in the adhesive composition shifts the cohesive–adhesive balance toward cohesion (increasing the cohesive strength of the adhesive film), which results in a decrease in the adhesion value, as presented in Figure 7. Improved cohesion of the adhesive layer caused by the addition of filler reduces peel adhesion. The palygorskite MPTMS modification showed no similar dependence. This proves the better compatibility of the first filler modification with the SI-PSA polymer matrix.

**Figure 9:**
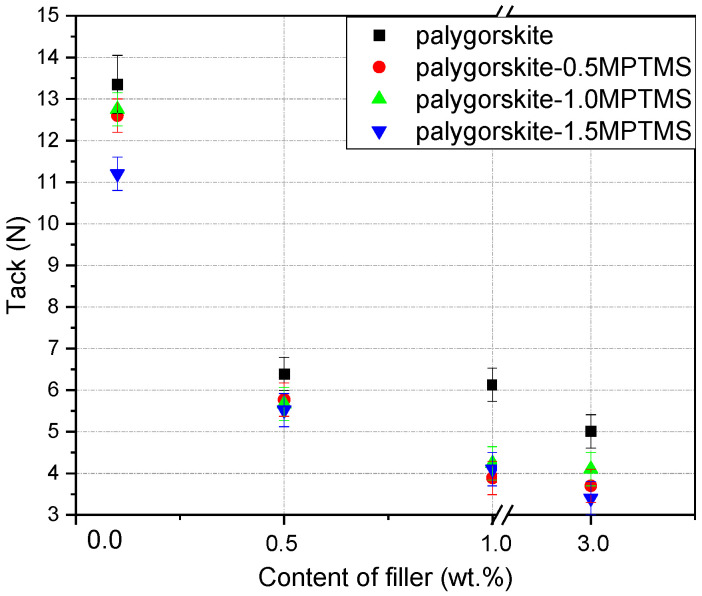
Influence of palygorskite modified by MPTMS addition on Si-PSA tape tack.

Both the unmodified palygorskite and its modifications had a standard effect on the tack values, lowering them—as the filler content increased, the tack value decreased (Figure 9). The degree of filler modification did not significantly affect the changes in these values [52,53]. A steady decrease in tack value was shown by samples containing the lowest filler values (0.1 wt.%) modified with 1.5 MPTMS. The same effect as for adhesion was not noted here; it may indicate the influence of the filler on the adhesive–cohesive balance and its better stabilization.

**Figure 10:**
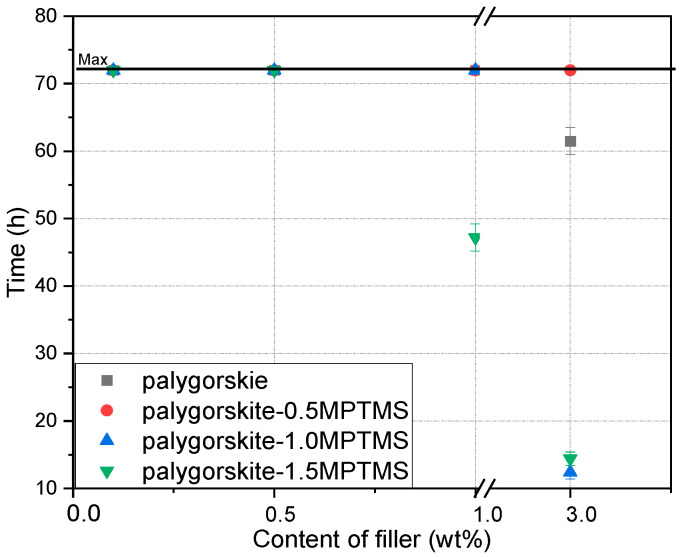
Influence of palygorskite modified by MPTMS addition on Si-PSA tape cohesion at 20 °C.

Figure 10 shows the results of the cohesion at 20 °C depending on the amount of the filler. In the case of palygorskite-modified MPTMS-modified filler, the cohesion did not last up to 72 h for fillings from 1.0 wt.%.

**Figure 11:**
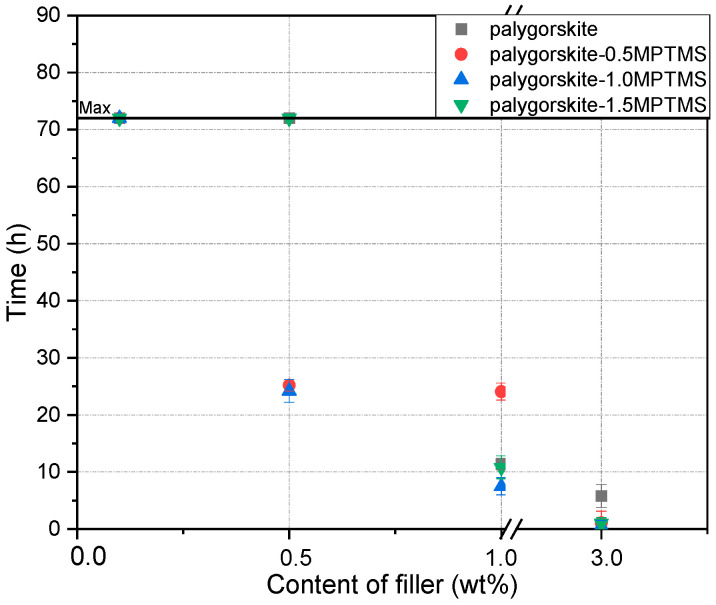
Influence of palygorskite modified by MPTMS addition on Si-PSA tape cohesion in 70 °C.

In the case of cohesion testing at elevated temperature (70 °C) only for small fillings (0.1 wt.%), the samples showed good cohesion for palygorskite modifications (Figure 11); this is due to the good compatibility of the filler with the resin and the stabilization of the adhesive–cohesive balance. In the case of higher fillings, the adhesive–cohesive balance was most likely shifted toward cohesion, so the samples did not withstand for 72 h.

**Figure 12:**
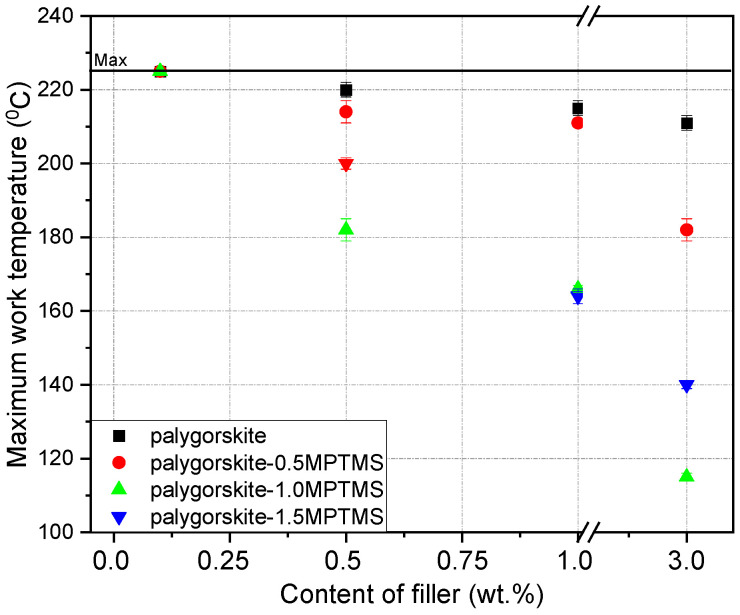
Influence of palygorskite modified by MPTMS addition on Si-PSA tape SAFT.

The results described translate directly into maximum work temperature measured in the SAFT test (Figure 12), where the maximum temperature of measurements was reached for the lower filling; in other cases it was lower, which, combined with the results from other tests, means that such a large addition has a negative effect on the properties of self-adhesive tapes. It is most likely caused by the partial agglomeration of the filler, shifting the adhesive–cohesive balance toward cohesion for highly filled adhesives with increased internal cohesion of the adhesive film. A small amount of filler necessary to achieve high thermal resistance and maintain the usefulness of the adhesive tape itself is a phenomenon confirmed in the literature [48,65,66].

**Figure 13:**
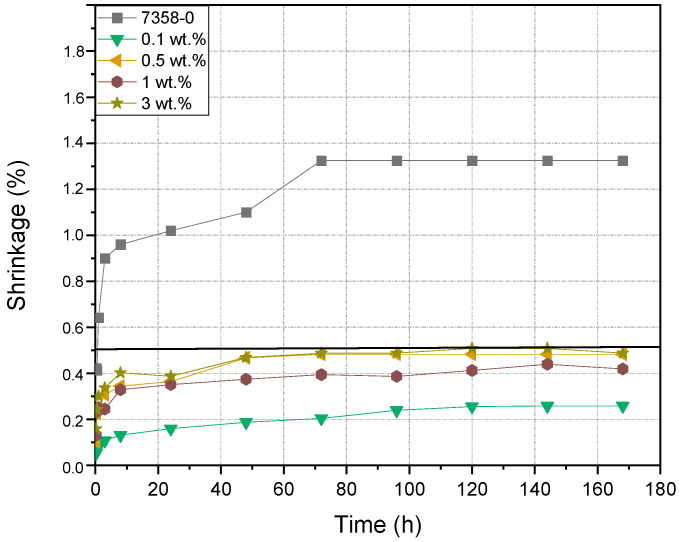
Influence of palygorskite addition on shrinkage Si-PSA.

**Figure 14:**
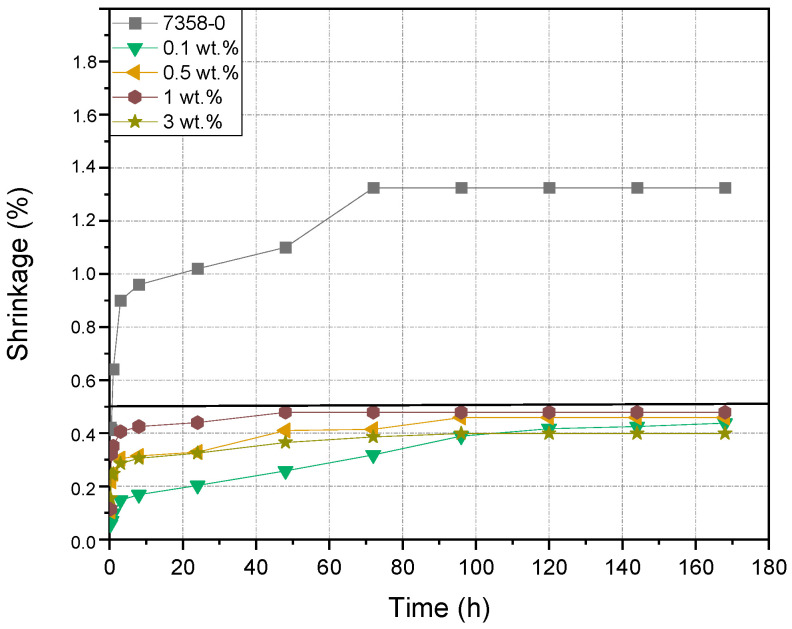
Influence of palygorskite-0.5MPTMS addition on shrinkage Si-PSA.

**Figure 15:**
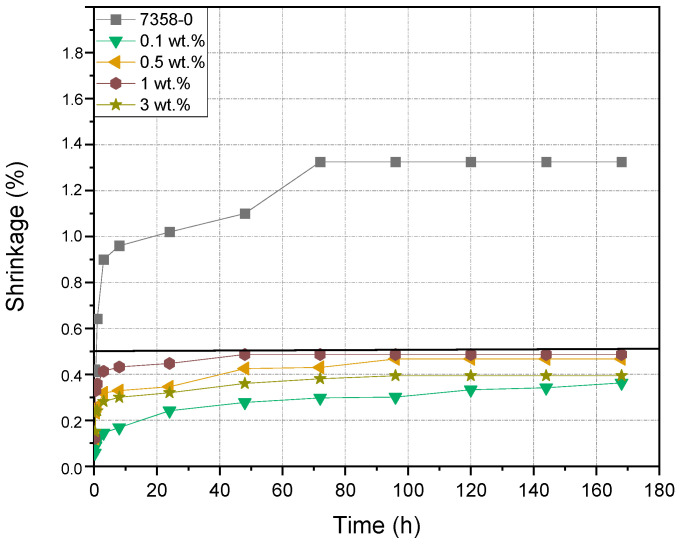
Influence of palygorskite-1.0MPTMS addition on shrinkage Si-PSA.

**Figure 16:**
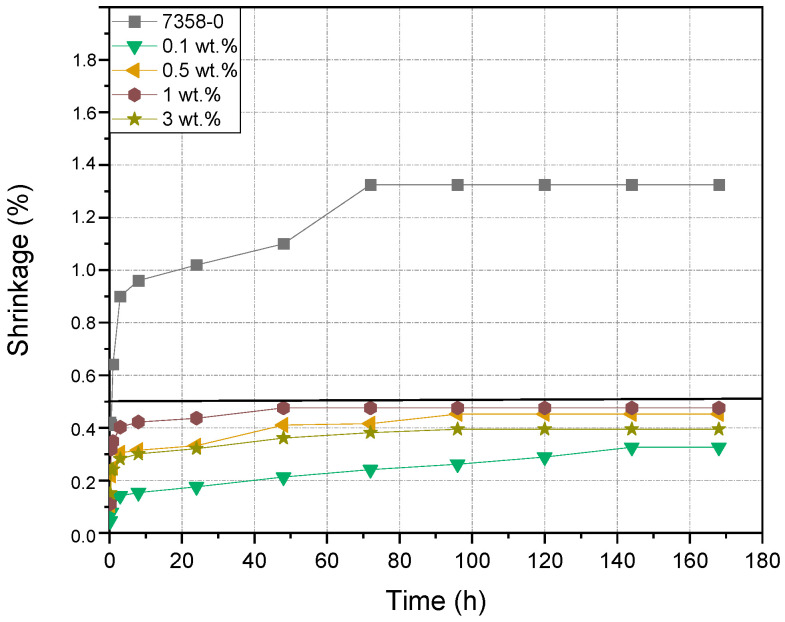
Influence of palygorskite-1.5MPTMS addition on shrinkage Si-PSA.

Figure 13, Figure 14, Figure 15 and Figure 16 present shrinkage increase with adhesive films, with and without fillers. In each case of filling, the reference was the adhesive film without filler, which exceeded the allowed 0.5% resistance to shrinkage. For each tested filler, tapes containing 0.1 wt.% showed the best effect filler; they achieved a stable shrinkage between about 0.2 to 0.5%. Higher fillings did not keep the shrinkage low, which could be caused by the partial agglomeration of the filler in the adhesive composition; nevertheless, none of the fillings achieved a stable shrinkage above the 0.5% limit. The stabilization effect of the cross-linked adhesive film obtained by adding fillers to the adhesive composition is an expected effect, but it is usually associated with higher fill concentrations, where the effect is obtained by saturation of places between the polymer chains and the impossibility of their free movement (the effect is visible with the increase in the amount of filler from 0.5 to 3 wt.%); in the case of low fillings (0.1 wt.%), the addition of a filler affects the density of the self-adhesive adhesive structure and thus increases its resistance to shrinkage due to the lack of their agglomeration [51,52].

## 4. Conclusions

Studies on the functionalization of calcined palygorskite were carried out by the dried method and by using the coupling agent 3-mercaptopropyl-trimethoxysilane (MPTMS) in amounts ranging from 0.5 to 1.5 wt.% relative to the amount of palygorskite, which was used to improve the chemical compatibility of palygorskite with specific resins for application in silicone pressure-sensitive adhesives. FTIR, TGA, and CHNS analysis results verified the effectiveness of the outlined MPTMS palygorskite modification procedure. According to this research, using the fibrous clay mineral palygorskite as a filler in the self-adhesive tape is feasible.

At the same time, the new tape maintains the cohesion, adhesion, tack, and shrinkage (basic functional properties) above acceptable levels for self-adhesive tapes, respectively, >72 h; 10 N25 mm^−1^; 8 N, and <0.5% [4]. Tapes containing 0.1 wt.% filler showed the best results while maintaining cohesion at room temperature and an elevated temperature level for >72 h, also demonstrating stable shrinkage at a level of 0.4% and exhibiting thermal resistance above 225 °C. The main difference between the tapes obtained was the value for adhesion and tack at levels of approximately 11–15 N25 mm^−1^ and 10–14 N. This proves that several types of self-adhesive tapes can be produced from the materials tested.

In addition, the surface modification resulted in the fact that, with a minimal decrease in performance compared to the unmodified filler, the addition of the modified filler improved the processing of plastics, which can be seen especially when the viscosity of analogous fillings is reduced. These results suggest that the clarity of the process will be facilitated and, in the future, the compositions will be efficient and stable at an industrial scale.

The self-adhesive product (tapes) exhibited increased thermal resistance and can be used as insulating materials in heating and construction works (when installing fireplaces); glass varnishing; masking in powder coating (baking in furnaces); and thermoprinting or electrical engineering in the insulation of motor windings.

## Figures and Tables

**Table 1:** The content of nitrogen, carbon, hydrogen, and sulfur in the calcined and functionalization of palygorskite, and the reaction efficiency of functionalization.

	N (%)	C (%)	H (%)	S (%)	Reaction Efficiency (%)
palygorskite-calcined	0.00 ± 0.00	1.08 ± 0.02	1.44 ± 0.01	0.00 ± 0.00	-
palygorskite-0.5MPTMS	0.00 ± 0.00	1.60 ± 0.03	1.52 ± 0.02	0.44 ± 0.01	66.00 ± 0.00
palygorskite-1.0MPTMS	0.00 ± 0.00	1.51 ± 0.05	1.50 ± 0.05	0.42 ± 0.02	28.00 ± 0.20
palygorskite-1.5MPTMS	0.00 ± 0.00	1.50 ± 0.08	1.50 ± 0.07	0.42 ± 0.04	18.70 ± 0.22
* palygorskite-0.5MPTMS	0.00	0.85	0.15	0.75	
** palygorskite-1.0MPTMS	0.00	1.70	0.30	1.50	
*** palygorskite-1.5MPTMS	0.00	2.55	0.45	2.25	

* Theoretical content of N, C, H, and S derived from 0.5 wt.% MPTMS; ** theoretical content of N, C, H, and S derived from 1.0 wt.% MPTMS; *** theoretical content of N, C, H, and S derived from 1.5 wt.% MPTMS.

## Data Availability

Not applicable.

## Supplementary Materials

The following supporting information can be downloaded at: https://www.mdpi.com/article/10.3390/ma16052092/s1, Figure S1: GC-MS chromatogram of the reaction mixture during the functionalization of palygorskite with MPTMS (immediately after the introduction of reactants into the reactor); Figure S2: GC-MS chromatogram of the reaction mixture during the functionalization of palygorskite with MPTMS (after 24 h of functionalization).
