# Supplementary material for: Study of Applying Naturally Occurring Mineral Materials for Silicone Pressure-Sensitive Adhesives
Source: Materials (Basel). 2023 Mar 3;16(5):2092. doi: 10.3390/ma16052092 (PMC10004305; doi:10.3390/ma16052092)
Supplement: Supplementary file 1 [file materials-16-02092-s001.zip › materials-2185181-supplementary.pdf]

Supplementary Materials

The compound of the reaction mixture during the functionalization of palygorskite with MPTMS were analyzed using GC-MS with a TRACE GC series apparatus equipped with a VOYAGER mass detector and using a DB5 capillary column (30m x 0.25μm x μ0.5 m). The following separation parameters were used for the analysis: a helium flow of 1.0mL/min, detector voltage of 350 V, and sample chamber temperature of 240° C. The thermostat temperature was increased according to the following program: isothermal at 50° C for 1 min, increased at 8° C/min, isothermal at 260 °C for 5 min, then cooled to 50 °C. The volume of the dispensed sample was 0.1 μL.

Figure S1 presents the GC-MS chromatogram of the reaction mixture during the functionalization of palygorskite with MPTMS (immediately after the introduction of reactants into the reactor).

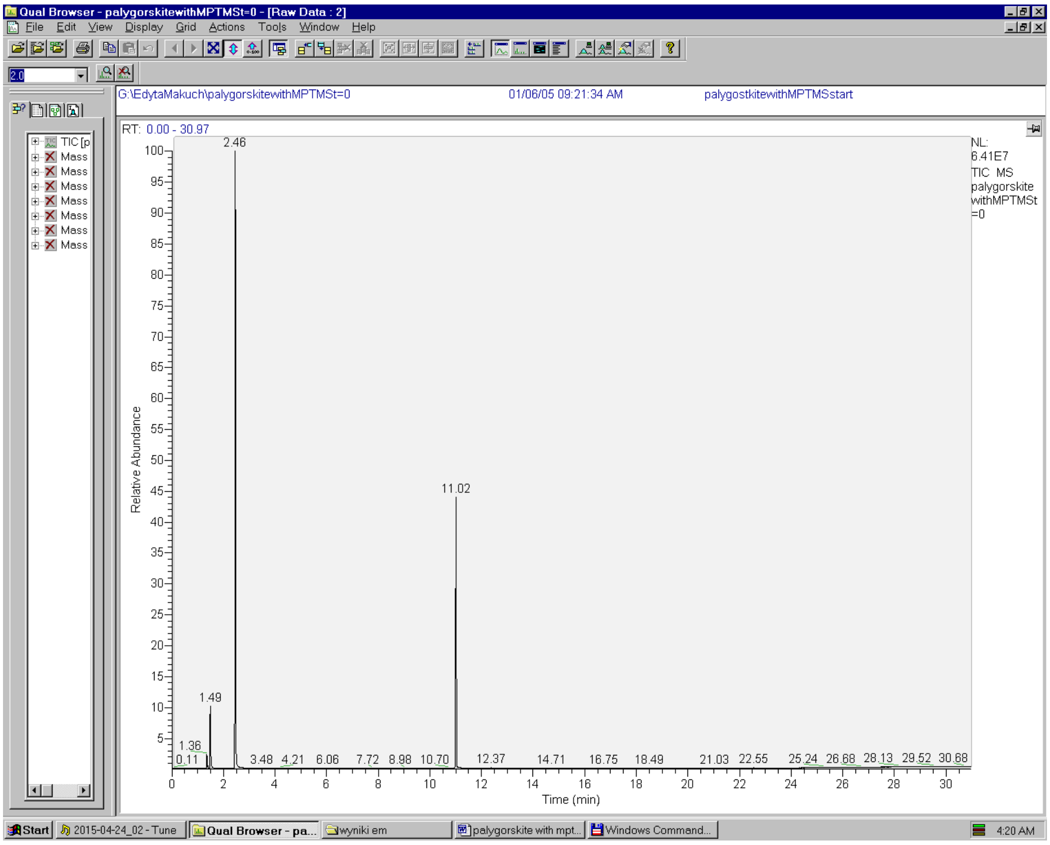

Figure S1. GC-MS chromatogram of the reaction mixture during the functionalization of palygorskite with MPTMS (immediately after the introduction of reactants into the reactor).

The following compounds were identified: methanol (RT=1.49 min), chloroform (RT=2.46 min), and MPTMS (RT=11.02 min) - Figure S1.

Figure S2 presents the GC-MS chromatogram of the reaction mixture during the functionalization of palygorskite with MPTMS (after 24 hours of functionalization).

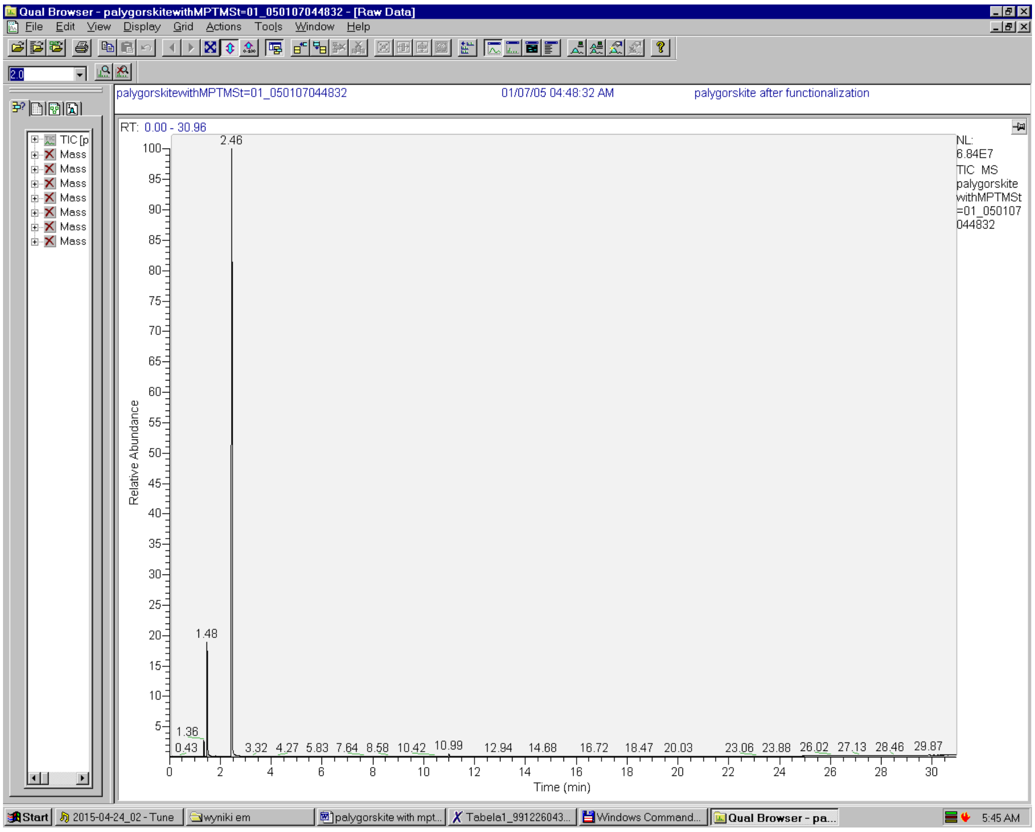

Figure S2. GC-MS chromatogram of the reaction mixture during the functionalization of palygorskite with MPTMS (after 24 hours of functionalization).

No MPTMS was observed in the reaction mixture after 24-hour palygorskite func-tionalization. In addition, the amount of methanol twice increased compared to the initial amount of methanol - Figure S2.
